# The impact of an anesthesia residency teaching service on anesthesia-controlled time and postsurgical patient outcomes: a retrospective observational study on 15,084 surgical cases

**DOI:** 10.1186/s13037-024-00394-z

**Published:** 2024-04-01

**Authors:** Davene Lynch, Paul D. Mongan, Amie L. Hoefnagel

**Affiliations:** 1 University of Florida College of Medicine, Jacksonville, USA; 2 University of Florida College of Medicine- Jacksonville, 655 West 8th Street, 32209 Jacksonville, FL Box C-72, USA

## Abstract

**Background:**

Limited data exists regarding the impact of anesthesia residents on operating room efficiency and patient safety outcomes. This investigation hypothesized that supervised anesthesiology residents do not increase anesthesia-controlled or prolonged extubation times compared to supervised certified registered nurse anesthetists (CRNA)/certified anesthesiologist assistants (CAA) or anesthesiologists working independently. Secondary objectives included differences in critical outcomes such as intraoperative hypotension, cardiac and pulmonary complications, acute kidney injury, and mortality.

**Methods:**

This retrospective single-center 24-month (January 1, 2020- December 31, 2021) cohort focused on primary outcomes of anesthesia-controlled times and prolonged extubation (>15 min) with additional assessment of secondary patient outcomes in adult patients having general anesthesia with an endotracheal tube or laryngeal mask airway for elective non-cardiac surgery. The study excluded sedation, obstetric, endoscopic, ophthalmology, and non-operating room procedures. Procedures were divided into three groups: anesthesiologists working solo, anesthesiologists supervising residents, or anesthesiologists supervising CRNA/CAAs. After univariate analysis, multivariable models were constructed to control for the univariate cofactor differences in the primary and secondary outcomes.

**Results:**

A total of 15,084 surgical cases met the inclusion criteria for this study for the three different care models: solo anesthesiologists (1,204 cases), anesthesiologist/resident pairing (3,146 cases), and anesthesiologist/CRNA/CAA (14,040 cases). Before multivariate analysis, the resident group exhibited longer anesthesia-controlled times (median, [interquartile range], 26.1 [21.7–32.0], *p* < 0.001), compared to CRNA/CAA (23.9 [19.7–29.5]), and attending-only surgical cases (21.0 [17.9–25.4]). After adjusting for covariates in a general linear regression model (age, BMI, ASA classification, comorbidities, arterial line insertion, surgical service, and surgical location), there were no significant differences in the anesthesia-controlled times between the provider groups. Prolonged extubation times (>15 min) were significantly less common in the anesthesiologist-only group compared to the other groups (*p* < 0.001). Despite these time differences, there were no clinically significant differences among the groups in postoperative pulmonary or cardiac complications, renal impairment, or the 30-day mortality rate of patients.

**Conclusion:**

Anesthesia residents do not increase anesthesia-controlled operating room times or adversely affect clinically relevant patient outcomes compared to anesthesiologists working independently or supervising certified registered nurse anesthetists or certified anesthesiologist assistants.

**Supplementary Information:**

The online version contains supplementary material available at 10.1186/s13037-024-00394-z.

## Background

Efforts to enhance operating room efficiency in the healthcare sector are integral to optimizing the use of valuable resources while maintaining patient safety. While resident involvement in surgical cases may increase operative times [1], some studies suggest that patient safety is not compromised by the presence of residents or fellows during surgical procedures [2–6]. In contrast, analysis of the 2005–2010 Surgeons National Surgical Quality Improvement Program database identified elevated complications (wound issues, pulmonary complications, and venous thromboembolism) in emergency procedures involving trainees [7]. Similar evaluations have evaluated the efficiency of anesthesia residents in practice but not outcomes [8–11]. In the United States, anesthesiology training spans four years, incorporating apprenticeship and didactic learning, with residents given increasing independence in recognition of their advanced knowledge and experience as they progress through training [12]. This process may be implicated in a 2–5 min delay from room entry to incision and concluding the procedure to room exit [8–11]. As such, it has been argued that residency training may impact operating room efficiency and potentially threaten patient safety if independence is given too soon [13]. 

In our institution, the initiation of anesthesiology orientation begins in the final six months of the internship (postgraduate year 1, PGY-1). This phase involves one-to-one pairing with a senior resident in a dedicated room for six weeks, supervised by an attending anesthesiologist and complemented by daily didactics. Once PGY-1 residents demonstrate competence after this initial period, they progress to the next stage of training, working under the supervision of staff anesthesiologists assigned to two rooms.

This retrospective review compares anesthesia-controlled operating room time among anesthesiologists working independently, collaborating with anesthesiology residents, or collaborating with certified registered nurse anesthetists (CRNA)/certified anesthesiologist assistants (CAA). Additionally, our secondary objectives involve assessing critical outcomes, including intraoperative hypotension (MAP < 55 and 65mmHg), cardiac and pulmonary complications, acute kidney injury, and mortality within these groups. Through this analysis, we aim to contribute valuable insights into the impact of anesthesia residents on efficiency and patient outcomes in the operating room setting.

## Methods

**Fig. 1:**
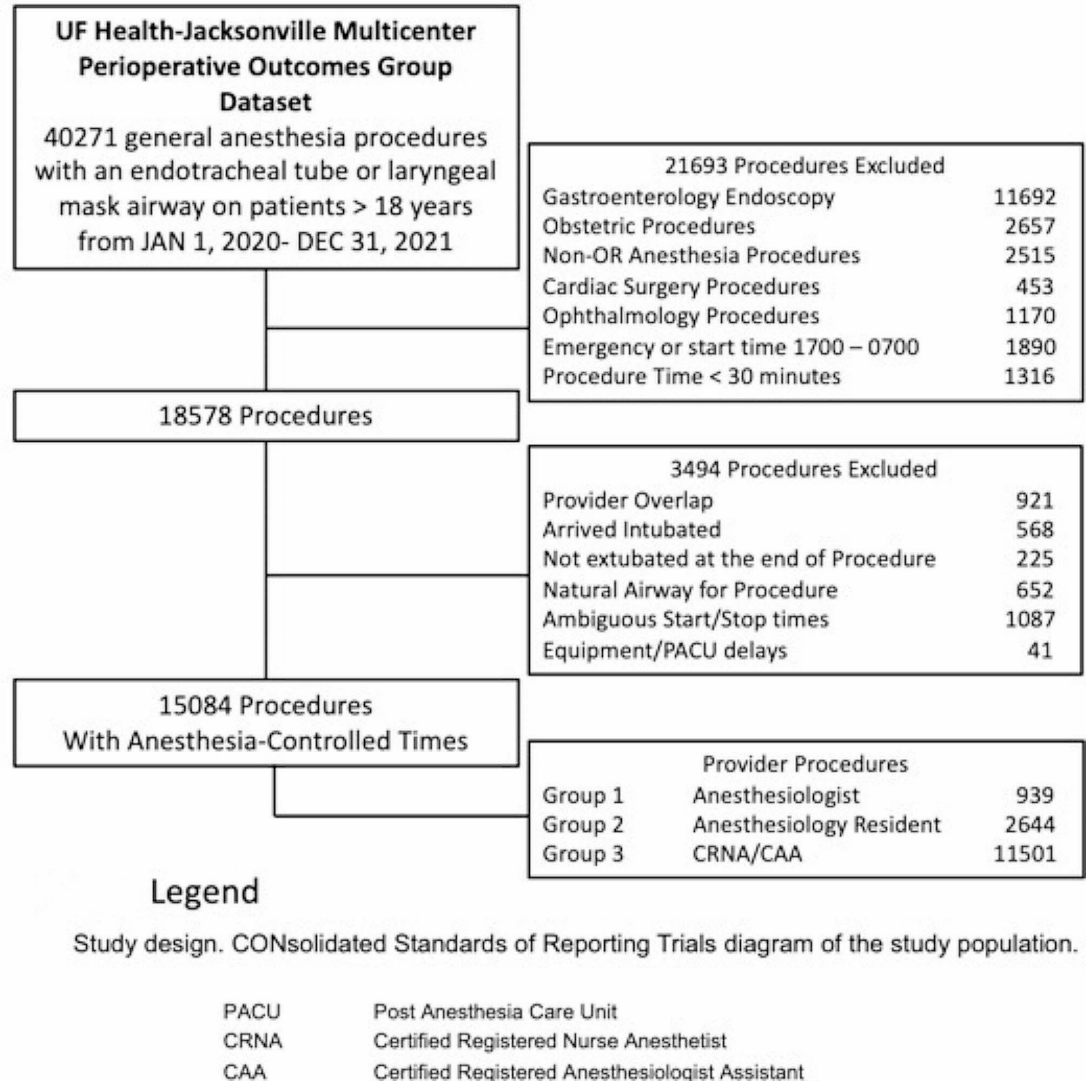
Consolidated standards of reporting trials diagram of the study population. *Figure legend*PACU - Post Anesthesia Care Unit, CRNA - Certified Registered Nurse Anesthetist, CAA - Certified Anesthesiologist Assistant

In this retrospective observational study, using UF Health-Jacksonville specific Multicenter Perioperative Outcomes Group data, we examined 24 months (January 1, 2020– December 31, 2021) of elective general anesthesia cases (endotracheal tube or laryngeal mask airway) and procedure times > 30 min duration (Monday-Friday starting between 0700 and 1700). Ethics approval was obtained from the University of Florida Institutional Review Board with a waiver of informed consent, Institutional Review Board, #201,800,667, September 12, 2022. The methodology for acquiring, validating, and mapping local electronic health record data to the Multicenter Perioperative Outcomes Group concepts, Elixhauser comorbidities, outcomes, and phenotype algorithms have been previously described [14–16]. We prospectively excluded all cases performed primarily with sedation or regional anesthesia. We also excluded cardiac, obstetric, pediatric (age < 18), endoscopic, ophthalmology, and non-operating room procedures and other exclusions as outlined in [Fig. 1].

The remaining procedures were divided into three groups: anesthesiologists working solo (Group 1), anesthesiologists supervising residents, and anesthesiologists supervising certified registered nurse anesthetists/certified anesthesiologist assistants (Groups 2 and 3, respectively). Procedures attributed to each group had the entire anesthetic delivered by that provider group. Cases involving anesthesiology residents were staffed 1:2 with a supervising anesthesiologist, and the supervising certified registered nurse anesthetists/certified anesthesiologist assistants (CRNA/CAA) group was staffed at 1:2 or 1:3. Anesthesiologists were present for induction and emergence (as indicated) for all cases. We compared anesthesia-controlled times (defined by the Anesthesia Clinical Directors Glossary of Times) from patient in-room to completion of induction and anesthesia-related procedures (anesthesia ready time) and end of the procedure to extubation and out-of-operating room times (out of-room time) for these three groups [17]. The total anesthesia-controlled time is the sum of anesthesia ready and out-of-room times. Anesthesia records with anesthesia ready or out-of-room time over 30 min were reviewed. They were excluded if there were documented non-anesthesia-related delays (procedural equipment issues, overlapping surgical cases, post-anesthesia care unit delays). The primary time-based outcome was the total anesthesia-controlled time. The definition of a prolonged extubation time (surgery end to extubation) was >15 min [18]. Secondary patient outcomes were derived from the Multicenter Perioperative Outcomes Group phenotypes (30-day mortality and significant outcome variables such as acute kidney injury, hypotension, length of stay, cardiac complications, and pulmonary complications).

Acute Kidney Injury (AKI) classification in the Multicenter Perioperative Outcomes Group dataset is based on serum creatinine criteria (stage 1 AKI, creatinine increases of 0.3 mg/dl or greater within 48 h or 1.5 to 1.9 times baseline within first seven days after surgery; stage 2 AKI, creatinine rise of 2.0 to 2.9 times baseline within seven days after surgery; and stage 3 AKI, creatinine rise to 4.0 mg/dl or greater or 3.0 times baseline). This classification is adopted from Kidney Disease-Improving Global Outcomes [16, 19]. Procedures with missing creatinine values for determining AKI stage and non-index procedures on the same patient (to avoid clustering and overcounting patient outcomes due to repeated procedures) were also excluded from the patient AKI outcome analysis.

The Multicenter Perioperative Outcomes Group uses the International Classification of Diseases (Ninth and Tenth Revision) discharge diagnosis codes to classify outcomes.

A pulmonary complication is derived from over 50 ICD 10 codes documented from the day of surgery through 90 days after surgery. These complications can be grouped into three general categories - respiratory failure, pneumonia, and pulmonary embolism. Cardiac complications are derived from ICD 10 discharge codes for myocardial infarction and cardiac arrest. Mortality, pulmonary, and cardiac complications were also limited to index procedures to reduce the clustering of infrequent outcomes for multiple surgical procedures.

Patient covariates extracted from the UF Health-Jacksonville Multicenter Perioperative Outcomes Group database included age, gender, race/ethnicity, body mass index (BMI), American Society of Anesthesiologists (ASA) physical status classification, ASA base units associated with the procedure, and the Multicenter Perioperative Outcomes Group defined preoperative comorbidities (defined by ICD-9/10 codes following the Elixhauser collation). The surgical service performing the procedure and the surgery location (community hospital, surgery center, and medical center) were also included in the data extraction in addition to the defined anesthesia and procedural times, and the Multicenter Perioperative Outcomes Group defined patient outcomes.

### Statistical analysis

Categorical variables are expressed as frequencies and percentages, and continuous variables are reported as the mean with standard deviation (+/- SD) or the median and interquartile range [25th-75th percentile]. As described above, descriptive statistics were used to examine the cohort’s demographics and clinical, surgical, and treatment characteristics, stratified by anesthesia provider groups. Categorical variables were compared using chi-squared tests. ANOVA or Kruskal–Wallis tests were used to compare continuous variables, depending on the distribution characteristics of the variable. As time-related data in healthcare often have rightward skewed distributions, logarithmic transformation was applied so the time data more closely approximated a normal distribution before making comparisons.

For the anesthesia-controlled time data, a general linear regression model was employed to determine if the association of provider groups with the total anesthesia-controlled time was influenced by significant covariates such as patient factors, surgical location (community hospital, surgery center, and medical center), and service variation. A binary logistic model was used to evaluate the impact of significant patient covariates, surgical procedure location, and service on the association of provider groups with categorical postoperative outcomes.

This review was an exploratory evaluation; no formal statistical power calculation was conducted before the study. A 24-month period was chosen to account for covariate analysis for low-incidence outcome variables such as pulmonary and cardiac complications and mortality [20, 21]. The statistical and data analysis plans were defined before accessing the data.

The data were analyzed using SPSS 29. A p-value less than 0.05 was considered statistically significant, and p-values were adjusted to account for multiple group comparisons. All results are reported according to the RECORD extension of the STROBE (STrengthening the Reporting of OBservational studies in Epidemiology) guidelines [22]. 

## Results

Our initial extract included 40,271 surgical cases from 2020 to 2021 (Fig. 1). Several exclusions were applied to the dataset, including endoscopy, obstetric, non-operating room, cardiac, and ophthalmology cases. After these exclusions, a total of 18,578 procedures remained for analysis. Further exclusions were made for cases with provider overlap, patients intubated on arrival or not extubated at the end of the procedure, cases with natural airways, cases with ambiguous start/stop times, and cases with documented equipment or post-anesthesia care unit (PACU) delays. The final dataset consisted of 15,084 surgical cases.

The breakdown of procedures by anesthesia provider group was as follows: 939 were performed by solo anesthesiologists, 2644 with attending/resident pairing, and 11,501 with attending/CRNA/CAA pairing. Residents were grouped as a single cohort as no significant differences were observed within the resident cohort based on clinical anesthesia year (CA 1–3) for anesthesia ready time, out-of-room time, total anesthesia-control time, and prolonged out-of-room times (Table 1).

**Table 1:** Induction, Extubation, and Anesthesia-Controlled times for Anesthesia Residents

	CA-1 *N* = 1436	CA-2 *N* = 906	CA-3 *N* = 802	*p*-value
Anesthesia Ready Time (min)	10.5 [8.4–13.3]	10.3 [8.5–13.5]	10.6 [8.5–13.5]	0.95
Surgery End to Extubation Time (min)	9.3 [6.9–12.7]	9.3 [6.7–12.8]	9.4 [7.0–13.0]	0.93
Prolonged Extubation, (>15 min, %)	5.2%	5.4%	5.9%	0.47
Surgery End to Out of Room Time (min)	14.9 [11.8–19.0]	15.1 [12.0–19.5]	15.0 [11.6–19.6]	0.95
Anesthesia-Controlled Time (min)	26.1 [21.7–31.8]	26.3 [22.0–31.9]	26.1 [21.2–32.8]	0.94

All cases with an Anesthesiology Resident were performed with a supervising Anesthesiologist who was required to be present for induction and extubation (as indicated) for all cases

Data are presented as median [25th percentile-75th percentile], or percent

Data were analyzed using SPSS 29 with a one-factor ANOVA, Kruskal-Wallis, or a Chi-Square test. P-values were adjusted to account for multiple comparisons between groups

min minutes

Table 2 presents patient demographics and associated comorbidities for the different anesthesia provider groups. While statistically significant, the clinical relevance of differences in age and BMI among the three groups is limited. There were significant differences in the racial distribution of patients, with a higher percentage of Black individuals in the resident cohort. This difference can be attributed to hospital location, with a higher percentage of Black patients at the Medical Center (37.9%) than the community hospital (29.2%). The resident and CRNA/CAA groups had a higher incidence of cardiovascular, pulmonary, vascular, and renal comorbidities than the anesthesiologist group. The ASA status was also higher for the resident and CRNA/CAA groups (3 [2 - 3]) compared to the anesthesiologist solo group (2 [2 - 3], *p* < 0.001), reflecting differences in comorbidities.

**Table 2:** Demographics and Co-Morbidities for anesthesia provider groups

	Anesthesiologist Group 1 *N* = 939	Resident Group 2 *N* = 2644	CRNA/CAA Group 3 *N* = 11,501	*p*-value
Age (years)	51.8 ± 15.8	50.4 ± 15.9	50.7 ± 16.0	0.07
BMI (kg/m^2^)	30.4 ± 7.4^**†**^	29.5 ± 7.8	29.6 ± 7.5	< 0.001
ASA score	2 [2 - 3]^**†**^	3 [2 - 3]	3 [2 - 3]	< 0.001
GENDER (% male)	41.0%	45.2%	45.1%	0.91
Race (%)				< 0.001
American Indian/Alaska Native	0.4%	0.1%	0.2%	
Asian/Pacific Islander	1.2%	0.9%	1.2%	
Black	29.2%	37.9%*	32.9%	
Other/Unknown	5.8%	6.7%	7.1%	
White	63.3%	54.4%*	58.7%	
Anesthesia Base Units	5.4 ± 1.8	5.3± 2.4	5.4 ± 2.3	0.74
Elixhauser Co-Morbidities ^a^				
CAD (%)	8.9%	10.3%	9.3%	0.176
Cardiac Arrythmia (%)	5.1%^**†**^	13.7%	12.2%	< 0.001
Chronic Pulmonary Disease (%)	7.5%^**†**^	13.7%*	10.9%	< 0.001
Congestive Heart Failure (%)	3.2%^**†**^	5.5%	4.6%	0.009
HTN Complicated (%)	5.8%^**†**^	10.2%	8.4%	< 0.001
HTN Uncomplicated (%)	22.3%^**†**^	30.1%*	26.0%	< 0.001
Diabetes Complicated (%)	2.7%	4.4%	3.8%	0.18
Diabetes Uncomplicated (%)	12.4%	12.0%	11.5%	0.48
Peripheral Vascular Disease (%)	2.0%^**†**^	5.8%*	4.1%	< 0.001
Renal Insufficiency (%)	5.9%	9.3%*	7.7%	< 0.001

All cases with an Anesthesiology Resident or CRNA/CAA were performed with a supervising Anesthesiologist who was required to be present for induction and extubation (as indicated) for all cases

Data are presented as mean ± standard deviation, median [25th percentile-75th percentile], or percent

Data were analyzed using SPSS 29 with a one-factor ANOVA, Kruskal-Wallis, or a Chi-Square/Fischer Exact test. P-values were adjusted to account for multiple comparisons between groups

† = *p* < 0.05 comparing Anesthesiologist group data to Resident and CRNA/CAA group data

* = *p* < 0.05 comparing Resident group data to Anesthesiologist and CRNA/CAA group data

^a^ = Comorbidity measures– extracted from Multi-Center Perioperative Outcomes Group dataset

BMI Body mass index

CRNA certified registered nurse anesthetist

CAA certified anesthesiologist assistant

CAD coronary artery disease

HTN hypertension

Table 3 summarizes perioperative procedure characteristics, including surgical location, service category, and procedural times. The surgical locations are a community hospital practice, the medical center at UF Health-Jacksonville, and a free-standing ambulatory surgery center. Of the anesthesiologist-only cases, 93.5% were done at the community hospital, 5.4% at the medical center, and 1.2% at the ambulatory surgery center. In contrast, the care team procedures were done predominantly at the medical center for residents (93.3%) and CRNA/CAA (67.1%). The breakdown of surgical service categories shows that anesthesiologist-only care was more common in general/plastics and gynecology and less likely for head and neck, acute care surgery, thoracic, and vascular procedures. Residents were assigned significantly more thoracic, vascular, and acute care surgery cases than the CRNA/CAA or anesthesiologists-only groups. The remainder of the surgical services were comparable between the groups.

**Table 3:** Perioperative Characteristics– Location, Surgical Service, Induction, Extubation, and Anesthesia-Controlled Times for the Anesthesia Provider Groups

	Anesthesiologist Group 1 *N* = 939		Resident Group 2 *N* = 2644	CRNA/CAA Group 3 *N* = 11,501	*p*-value
Surgical Location					< 0.001
Medical Center, (%, n)	5.4% (51) ^**†**^		93.3% (2467) *	67.1% (7717)	
Ambulatory Surgery Center, (%, n)	1.2% (11) ^**†**^		2.9% (77) *	8.6% (992)	
Community Hospital, (%, n)	93.5% (877) ^**†**^		3.8% (100) *	24.3% (2792)	
Surgical Service					< 0.001
General/Plastics, (%, n)	27.3% (256) ^†^		21.1% (558)	20.2% (2328)	
Gynecology, (%, n)	21.5% (202) ^†^		12.8% (339)	13.0% (1492)	
Head and Neck, (%, n)	4.2% (39) ^†^		14.0% (370)	15.0% (1725)	
Neurosurgery, (%, n)	4.3% (40)		4.9% (130)	6.2% (717)	
Orthopedics, (%, n)	33.5% (315)		29.0% (768)	31.3% (3577)	
ACS, Thoracic, Vascular, (%, n)	2.0% (19) ^†^		11.0% (291)*	6.2% (716)	
Urology, (%, n)	7.2% (68)		7.1% (188)	8.2% (946)	
Endotracheal tube insertion %	69.6%		71.2%	68.7%	0.07
Arterial Line Insertion %	1.2%^**†**^		10.7%*	9.3%	< 0.001
Surgical Times					
Anesthesia Ready Time (min)	8.7 [7.0-10.7]^**†**^		10.5 [8.5–13.4]*	9.5 [7.7–12.0]	< 0.001
Surgery End to Extubation Time (min)	7.9 [6.0-10.3]^**†**^		9.4 [6.8–12.8]*	9.0 [6.6–12.3]	< 0.001
Prolonged Extubation Time (>15 min, %, n)		1.7% (20) ^**†**^	5.4% (170)	4.5% (631)	< 0.001
Surgery End to Out of Room Time (min)	12.1 [9.5–15.1]^**†**^		14.9 [11.7–19.3]*	13.9 [10.8–18.0]	< 0.001
Anesthesia-Controlled Time (min)	21.0 [17.9–25.4]^**†**^		26.1 [21.7–32.0]*	23.9 [19.7–29.5]	< 0.001
Anesthesia Ready to procedure start (min)	21.6 [16.0-27.5]^**†**^		24.0 [17.8–32.7]	23.0 [16.9–31.8]	< 0.001
Procedure Time (min)	79.0 [41.0-121.0]^**†**^		90.0 [49.0-148.0]	89.0 [48.0-150.0]	< 0.001
Total time in OR (min)	122.3 [79.3-168.4]^**†**^		137.3 [91.3-204.4]	135.8 [89.2-203.3]	< 0.001

All cases performed with an Anesthesiology Resident or CRNA/CAA were performed with a supervising Anesthesiologist that was required to be present for induction and extubation (as indicated) for all cases

Data are presented as mean ± standard deviation, median [25th percentile-75th percentile], or percent

Data were analyzed using SPSS 29 with a one-factor ANOVA, Kruskal-Wallis, or a Chi-Square test. P-values were adjusted to account for multiple comparisons between groups

† = *p* < 0.05 comparing Anesthesiologist group data to Resident and CRNA/CAA group data

* = *p* < 0.05 comparing Resident group data to Anesthesiologist and CRNA/CAA group data

ACS acute care surgery

CRNA certified registered nurse anesthetist

CAA certified anesthesiologist assistant

min minutes

OR operating room

Compared to the anesthesiologist or CRNA/CAA groups, the resident group had longer anesthesia-ready times, 10.5 [8.5–13.4] vs. 9.5 [7.7–12.0] for the CRNA/CAA group and 8.7 [7.0-10.7] minutes for the anesthesiologist only group (Table 3, *p* < 0.001). Similar trends were seen in the surgery end-to-extubation and out-of-room times– resident, 14.9 [11.7–19.3], CRNA/CAA, 13.9 [10.8–18.0], and anesthesiologist solo,12.1 [9.5–15.1] minutes, respectively (*p* < 0.001). Overall, anesthesia-controlled times were significantly shorter in the anesthesiologist-only group at 21.0 [17.9–25.4] compared to 23.9 [19.7–29.5] for the CRNA/CAA group and 26.1 [21.7–32.0] minutes in the resident group (*p* < 0.001). Procedural times, including anesthesia ready-to-procedure start, procedure time, and total time in the operating room, were significantly shorter in the anesthesiologist-only practice than in the care team model, reflecting the community hospital practice.(Table 3) However, analysis of the total anesthesia-controlled time by general linear regression analysis adjusted for covariates (age, BMI, ASA classification, comorbidities, arterial line insertion, surgical service, and surgical location) did not show a statistically significant difference between the provider groups for estimated marginal mean for the anesthesia-controlled times. (Supplemental Tables 1 A-D) Prolonged extubation times (>15 min) were significantly less common in the anesthesiologist-only group compared to the care team groups (*p* < 0.001). This difference was still present after binary logistic regression for the resident group compared to the anesthesiologist solo group.(Supplemental Table 1E).

Table 4 presents the clinical outcomes based on the anesthesia provider group. The occurrence and duration of decline in mean arterial pressure (MAP) below 55 or 65 mmHg were similar for all groups.

**Table 4:** Patient Outcomes

	Anesthesiologist Group 1 *N* = 939	Resident Group 2 *N* = 2644	CRNA/CAA Group 3 *N* = 11,501	*p*-value
MAP < 55mmHg, >15 min, (%, n)	2.8%, (26)	2.5%, (66)	2.7%, (310)	0.11
MAP < 55mmHg, > 1 min, (%, n)	29.7% (279)	30.4% (804)	28.6% (3289)	0.10
minutes > 1, MAP < 55mmHg	5 [3 - 9]	5 [2 - 9]	4 [2 - 8]	0.26
MAP < 65mmHg, >15 min, (%, n)	31.2%, (293)	31.9%, (843)	30.3%, (3485)	0.62
minutes >15, MAP < 65mmHg,	29 [21–44]	30 [22–45]	31 [21–44]	0.48
Cardiac Complication, (%, n)	0.2%, (2)	0.5%, (13)	0.5%, (56)	0.25
Myocardial Infarction, (%, n)	0.1%, (1)	0.2%, (5)	0.3%, (34)	0.56
Pulmonary Complications,				
Respiratory Failure, (%, n)	0.4% (4)	0.6% (17)	0.6% (75)	0.64
Pneumonia (%, n)	0.0% (0)	0.1% (4)	0.2% (23)	0.44
Pulmonary Embolus (%, n)	0.0% (0)	0.07% (2)	0.05% (6)	0.79
MPOG AKI Stage (n)	225	1231	5159	0.08
0, (%, n)	91.1% (205)	93.2% (1147)	91.8% (4735)	
1, (%, n)	6.2% (14)	5.7% (70)	6.3% (324)	
2, (%, n)	2.7% (6)	1.0% (72)	1.2% (64)	
3, (%, n)	0.0% (0)	0.2% (2)	0.7% (36)	
Length of Stay, days	0.0 [0.0–1.0] ^**†**^	1.0 [0.0–3.0]	0.0 [0.0–2.0]	< 0.001
30 Day Mortality, (%, n)	0.2% (2)	0.3% (8)	0.4% (46)	0.29

All cases performed with an Anesthesiology Resident or CRNA/CAA were performed with a supervising Anesthesiologist who was required to be physically present for induction and extubation (as indicated) for all cases

Data are presented as mean ± standard deviation, median [25th percentile-75th percentile], or percent

Data were analyzed using SPSS 27 with a one-factor ANOVA, Kruskal-Wallis, or a Chi-Square test. P-values were adjusted to account for multiple comparisons between groups

† = *p* < 0.05 comparing Anesthesiologist group data to Resident and CRNA/CAA group data

* = *p* < 0.05 comparing Resident group data to Anesthesiologist and CRNA/CAA group data

CRNA certified registered nurse anesthetist

CAA certified anesthesiologist assistant

MAP mean arterial pressure

Min minutes

AHRQ Agency for Healthcare Research and Quality

MPOG Multicenter Perioperative Outcomes Group

AKI acute kidney injury

A binary logistic regression analysis was performed to determine the effects of the significant univariate factors on the likelihood of a MAP < than 55 mmHg (> 1 min or > 10 min) or < 65 mmHg (>15 min). The Anesthesiologist group had slightly increased odds for low mean arterial pressures (1.2–1.7). The surgical location (medical center), surgical services, anesthesia duration, and various comorbidities (cardiac arrhythmia, pulmonary disease, complicated diabetes, and peripheral vascular disease) were associated with increased odds of low mean arterial pressure.(Supplemental Tables 2 A-C) The odds ratio effect sizes in the final models were generally small (OR < 1.86) [23, 24]. 

The univariate incidence of cardiac complications, pulmonary complications, and AKI were similar in all groups, as well as the 30-day mortality.(Table 4) Within the Multicenter Perioperative Outcomes Group dataset, a pulmonary complication comprises over 50 ICD 10 codes documented from the day of surgery through 90 days after surgery. These complications can be grouped into three general categories - respiratory failure, pneumonia, and pulmonary embolism. The univariate incidence of pulmonary complications was similar in the resident, CRNA/CAA, and anesthesiologist-only group. After logistic regression, the provider group and location had no significant impact on the pulmonary complications. (Supplemental Table 3)

Similarly, after binary logistic regression, the provider groups and locations had no increased odds of cardiac complications, myocardial infarction, or mortality, while age, anesthesia duration, and various comorbidities significantly impacted those outcomes. (Supplemental Tables 4–6)

After binary logistic regression, the odds ratio for AKI grade 1–3 remained similar for the provider groups with decreased odds of procedures performed at the ambulatory center. (Supplemental Table 7) Finally, the length of stay differences between provider groups was insignificant after adjusting for significant covariates and location. (Supplemental Tables 8 A-C)

## Discussion

In this retrospective cohort study, we investigated the efficiency and clinical outcomes of anesthesia care provided by supervised residents, supervised CRNA/CAA teams, and solo-practicing anesthesiologists and compared those findings to previous literature [9–11, 25, 26]. Although small differences in anesthesia-controlled times initially surfaced in univariate analysis, these distinctions disappeared after considering confounding variables in the multivariate analysis. Notably, when assessing clinically relevant outcomes like intraoperative hypotension, renal, pulmonary, or cardiac complications, and mortality, no significant differences were found among care provided by anesthesia residents, CRNA/CAAs supervised by anesthesiologists, or solo-practicing anesthesiologists.

Our univariate analysis identified statistically significant differences in induction (1–2 min), emergence (2–3 min), and overall anesthesia-controlled time (3–5 min) between supervised resident/CRNA-CAA groups and solo-practicing anesthesiologists. These findings align with a previous study that found that a 2–3 min increase in the anesthesia-controlled time by first-year anesthesia residents, compared to solo anesthesiologists, may not significantly impact operating room efficiency [9]. This finding is consistent with a retrospective study by Urman et al. on ASA 1 and 2 patients having ambulatory surgery (*n* = 2427), where residents increased the anesthesia-controlled time by two minutes [26]. Schuster et al. also found a small increase (2–3 min) in induction times for residents compared to staff anesthesiologists in a study involving endotracheal tube and laryngeal mask airway placement (*n* = 241) [11]. However, no significant impact of provider groups on the anesthesia-controlled time was shown after logistic regression analysis, accounting for location, arterial line insertion, and differences in surgical services.

Despite the lack of multivariate analysis differences in the anesthesia-controlled time, prolonged emergence was higher in the resident group, with an incidence of 5.4% versus 4.5% and 1.2% in the CRNA/CAA group and solo anesthesiologists, respectively (*p* < 0.001). The factors influencing the anesthesia-controlled time and prolonged extubation times are complex, and specific anesthesia-related modifications can improve or detract from these measures [26]. Overall, the incidence of prolonged extubation times in every group in our cohort was lower than the 15% previously reported [18, 27]. House and Calloway et al.‘s retrospective evaluation of 7687 cases from an electronic database with residents providing anesthesia care found a 13.9% incidence of emergence times >15 min compared to 5.4% in our resident cohort [8]. The higher occurrence of prolonged time to extubation observed by previous evaluations [8, 18] may be due to changes in practice and the elimination of the routine use of isoflurane for maintenance of anesthesia and neostigmine for reversal of neuromuscular blockade in our practice. This is supported by a meta-analysis revealing that desflurane or sevoflurane instead of isoflurane could reduce prolonged extubation times by 95% and 91%, respectively [18, 28]. Other factors are that only 68% of our patients had endotracheal tubes with nearly 100% reversal of residual neuromuscular blockade with sugammadex, which may also influence the time to extubation [29]. 

In contrast to a previous study suggesting increased patient risks in the second year of anesthesia resident training, our cohort, analyzed through univariate and multivariate approaches, showed no statistically significant differences in kidney injury, post-surgical pulmonary or cardiac events, or 30-day mortality across three patient care models [13]. In addition, there were no group differences in the occurrence or duration of MAP decreases below 55 or 65 mmHg. The incidence and duration of intraoperative hypotension (MAP < 55 and 65 mmHg) observed in our cohort, a potentially modifiable risk factor associated with adverse outcomes, and the incidence of postoperative acute kidney injury in our study were aligned with findings of previously reported data [16, 30–34]. These findings indicate that anesthesia residents can provide care comparable to other provider groups when appropriately trained and supervised, dispelling concerns about heightened risks associated with resident training.

Our study has limitations, including potential bias from non-randomized case allocation, leading to variations in practice settings, surgical case mix, and comorbidities. Data modeling challenges can include numerous covariates coupled with a few rare events. (< 10 events per variable in multivariate models) [20, 21, 35]. We addressed these by ensuring a large sample size, excluding procedures performed primarily with regional anesthesia and/or sedation and other procedures introducing other statistical concerns (cardiac, non-operating room, and endoscopy procedures). We also limited multiple variable models to covariates, significantly contributing to the models. While physiologic variables within the Multicenter Perioperative Outcomes Group dataset are robust and highly validated as they are directly linked to automated data collection, there are potential errors in comorbidities and some outcomes due to coding data entry. Time-stamped human data entry may also have errors in time entry or be subject to interpretation. One time point subject to interpretation is anesthesia ready time (1.13 Time at which the patient has a sufficient level of anesthesia established to begin surgical preparation of the patient and remaining anesthetic chores do not preclude positioning and prepping the patient) [17]. This time point is inherent to the definition of anesthesia-controlled time [36]. In 90–99% of the cases in this cohort, it corresponds with the insertion of the endotracheal tube or laryngeal mask airway, while in the remainder of the cases, the anesthesia ready time includes additional time for other anesthetic procedures. Anesthesia-ready time and, thus, anesthesia-controlled time may also be favorably impacted by collaborative team care in some resident and CRNA/CAA cases. While never systematically studied, any timed data point error is probably measured in minutes. Retrospective data also pose generalizability issues due to changes in practice over time. We mitigated this internally by studying a stable 24-month period without major curriculum or anesthetic management changes, ensuring consistency in practice. This period was selected because of the decreased use of isoflurane after 2020 and the increased use of LMAs, glideslopes, and sugammadex.

## Conclusion

In this retrospective cohort, we conclude that anesthesia residents with appropriate orientation, training, and supervision do not significantly increase anesthesia-controlled operating room times. Moreover, there were no discernible differences in clinically relevant outcomes across the various patient care models. Despite these insights, the need for multicenter efforts to validate our findings and increase generalizability remains imperative, particularly in confirming secondary outcome findings related to intraoperative hypotension, cardiac and pulmonary complications, renal failure, and mortality.

### Electronic supplementary material

Below is the link to the electronic supplementary material.

Supplementary Material 1

## Data Availability

The datasets used during the current study are available from the corresponding author upon reasonable request.

## Abbreviations

CA: clinical anesthesia year
CRNA: certified registered nurse anesthetists
CAA: certified anesthesiologist assistants
BMI: body mass index
ASA: American Society of Anesthesiologists
PGY-1: postgraduate year 1
MAP: mean arterial pressure
AKI: Acute Kidney Injury
KDIGO: Kidney Disease-Improving Global Outcomes
SD: standard deviation
STROBE: Strengthening the reporting of observational studies in epidemiology
PACU: post-anesthesia care unit
OR: odds ratio
