# Supplementary material for: The impact of an anesthesia residency teaching service on anesthesia-controlled time and postsurgical patient outcomes: a retrospective observational study on 15,084 surgical cases
Source: Patient Saf Surg. 2024 Apr 1;18:12. doi: 10.1186/s13037-024-00394-z (PMC10985884; doi:10.1186/s13037-024-00394-z)
Supplement: Supplementary file 1 — Supplementary Material 1 [file 13037_2024_394_MOESM1_ESM.docx]

SUPPLEMENTAL TABLE 1A. General Linear Regression Model for Estimated Marginal Mean for Total ACT Time.

| Provider | Estimated Mean | Std. Error | 95% Confidence Interval | |
| --- | --- | --- | --- | --- |
|  |  |  | Lower | Upper |
| Anesthesiologist | 20.166 | 1.448 | 17.328 | 23.005 |
| CRNA/CAA | 20.279 | .557 | 19.189 | 21.370 |
| Resident | 21.244 | .383 | 20.494 | 21.995 |

General Linear Model in SPSS for the categorical variables of provider group and location with inclusion of the following significant covariates from TABLE 1 – Age, Total OR time, Body Mass Index, arterial line placement, surgical service, ASA status, Race, Cardiac Arrhythmia, Chronic Pulmonary Disease, Hypertension Complicated, Hypertension Uncomplicated, Peripheral Vascular Disorders, and Renal Insufficiency

SUPPLEMENTAL TABLE 1B. Pairwise Comparisons of Providers for the General Linear Regression Model for the Estimated Marginal Mean for Total ACT Time

| (I) Provider | (J) Provider | Mean Difference (I-J) | Std. Error | P value | 95% CI for Difference | |
| --- | --- | --- | --- | --- | --- | --- |
|  |  |  |  |  | Lower | Upper |
| Anesthesiologist | CRNA/CAA | -.113 | 1.551 | 1.000 | -3.828 | 3.601 |
|  | Resident | -1.078 | 1.498 | 1.000 | -4.664 | 2.508 |
| CRNA/CAA | Anesthesiologist | .113 | 1.551 | 1.000 | -3.601 | 3.828 |
|  | Resident | -.965 | .675 | .458 | -2.580 | .650 |
| Resident | Anesthesiologist | 1.078 | 1.498 | 1.000 | -2.508 | 4.664 |
|  | CRNA/CAA | .965 | .675 | .458 | -.650 | 2.580 |
|  | | | | | | |
| P value adjustment for multiple comparisons: Bonferroni. | | | | | | |

General Linear Model in SPSS for the categorical variables of provider group and location with inclusion of the following significant covariates from TABLE 1 – Age, Total OR time, Body Mass Index, arterial line placement, surgical service, ASA status, Race, Cardiac Arrhythmia, Chronic Pulmonary Disease, Hypertension Complicated, Hypertension Uncomplicated, Peripheral Vascular Disorders, and Renal Insufficiency

SUPPLEMENTAL TABLE 1C. Comparison of the General Linear Regression Model for the Estimated Marginal Mean for Total ACT Time stratified for location and provider.

| Provider | Location | Mean | Std. Error | 95% Confidence Interval | |
| --- | --- | --- | --- | --- | --- |
|  |  |  |  | Lower | Upper |
| Community Hospital | Anesthesiologist | 19.267 | .959 | 17.387 | 21.148 |
|  | CRNA/CAA | 19.197 | .257 | 18.693 | 19.702 |
|  | Resident | 20.580 | .830 | 18.952 | 22.208 |
| Ambulatory Center | Anesthesiologist | 20.849 | 2.198 | 16.541 | 25.157 |
|  | CRNA/CAA | 19.786 | 1.651 | 16.550 | 23.023 |
|  | Resident | 20.085 | .773 | 18.569 | 21.600 |
| Medical Center | Anesthesiologist | 20.280 | 3.333 | 13.747 | 26.813 |
|  | CRNA/CAA | 21.885 | .134 | 21.621 | 22.148 |
|  | Resident | 22.369 | .229 | 21.919 | 22.819 |

General Linear Model in SPSS for the categorical variables of provider group and location with inclusion of the following significant covariates from TABLE 1 – Age, Total OR time, Body Mass Index, arterial line placement, surgical service, ASA status, Race, Cardiac Arrhythmia, Chronic Pulmonary Disease, Hypertension Complicated, Hypertension Uncomplicated, Peripheral Vascular Disorders, and Renal Insufficiency

SUPPLEMENTAL TABLE 1D. Pairwise Comparisons of Providers by Location for the General Linear Regression Model for the Estimated Marginal Mean for Total ACT Time

| Location | (I) Provider | (J) Provider | Mean Difference (I-J) | Std. Error | P value | 95% CI for Difference | |
| --- | --- | --- | --- | --- | --- | --- | --- |
|  |  |  |  |  |  | Lower | Upper |
| Community Hospital | Anesthesiologist | CRNA/CAA | .226 | .987 | 1.000 | -2.138 | 2.590 |
|  |  | Resident | -1.270 | 1.275 | .958 | -4.323 | 1.783 |
|  | CRNA/CAA | Anesthesiologist | -.226 | .987 | 1.000 | -2.590 | 2.138 |
|  |  | Resident | -1.496 | .883 | .271 | -3.610 | .619 |
|  | Resident | Anesthesiologist | 1.270 | 1.275 | .958 | -1.783 | 4.323 |
|  |  | CRNA/CAA | 1.496 | .883 | .271 | -.619 | 3.610 |
| Ambulatory Center | Anesthesiologist | CRNA/CAA | 1.059 | 2.735 | 1.000 | -5.490 | 7.609 |
|  |  | Resident | .824 | 2.318 | 1.000 | -4.726 | 6.374 |
|  | CRNA/CAA | Anesthesiologist | -1.059 | 2.735 | 1.000 | -7.609 | 5.490 |
|  |  | Resident | -.235 | 1.814 | 1.000 | -4.580 | 4.109 |
|  | Resident | Anesthesiologist | -.824 | 2.318 | 1.000 | -6.374 | 4.726 |
|  |  | CRNA/CAA | .235 | 1.814 | 1.000 | -4.109 | 4.580 |
| Medical Center | Anesthesiologist | CRNA/CAA | -1.350 | 3.320 | 1.000 | -9.299 | 6.598 |
|  |  | Resident | -1.837 | 3.325 | 1.000 | -9.798 | 6.123 |
|  | CRNA/CAA | Anesthesiologist | 1.350 | 3.320 | 1.000 | -6.598 | 9.299 |
|  |  | Resident | -.487 | .254 | .167 | -1.096 | .122 |
|  | Resident | Anesthesiologist | 1.837 | 3.325 | 1.000 | -6.123 | 9.798 |
|  |  | CRNA/CAA | .487 | .254 | .167 | -.122 | 1.096 |

Based on estimated marginal means

P value adjustment for multiple comparisons: Bonferroni.

General Linear Model in SPSS for the categorical variables of provider group and location with inclusion of the following significant covariates from TABLE 1 – Age, Total OR time, Body Mass Index, arterial line placement, surgical service, ASA status, Race, Cardiac Arrhythmia, Chronic Pulmonary Disease, Hypertension Complicated, Hypertension Uncomplicated, Peripheral Vascular Disorders, and Renal Insufficiency

SUPPLEMENTAL TABLE 1E. Final factors included in the binary logistic regression models for prolonged extubation times > 15 min.

|  | | Wald | df | P value | OR | 95% CI for OR | |
| --- | --- | --- | --- | --- | --- | --- | --- |
|  |  |  |  |  |  | Lower | Upper |
|  | Provider |  |  |  |  |  |  |
|  | Resident (reference) | 8.843 | 2 | .012 |  |  |  |
|  | Anesthesiologist | 8.433 | 1 | .004 | .608 | .434 | .850 |
|  | CRNA/CAA | 2.696 | 1 | .101 | .897 | .788 | 1.021 |
|  | Location |  |  |  |  |  |  |
|  | Community Hospital (reference) | 71.678 | 2 | <.001 |  |  |  |
|  | Ambulatory Center | 43.236 | 1 | <.001 | 2.091 | 1.679 | 2.606 |
|  | Medical Center | 65.425 | 1 | <.001 | 1.879 | 1.613 | 2.190 |
|  | Surgical Service |  |  |  |  |  |  |
|  | Gynecology (reference) | 310.999 | 6 | <.001 |  |  |  |
|  | Neurosurgery | 95.573 | 1 | <.001 | 3.016 | 2.417 | 3.763 |
|  | General/Plastics | .013 | 1 | .909 | 1.011 | .844 | 1.210 |
|  | Head and Neck | 66.210 | 1 | <.001 | 2.083 | 1.746 | 2.486 |
|  | Orthopedics | 8.418 | 1 | .004 | .769 | .644 | .918 |
|  | Urology | .000 | 1 | .999 | 1.000 | .770 | 1.299 |
|  | ACS, Thoracic, Vascular | 1.008 | 1 | .315 | 1.125 | .894 | 1.417 |
|  | Anesthesia Duration | 193.265 | 1 | <.001 | 1.003 | 1.003 | 1.004 |
|  | Cardiac Arrythmia | 16.479 | 1 | <.001 | 1.351 | 1.169 | 1.563 |
|  | Constant | 640.419 | 1 | <.001 | .049 |  |  |

Provider, Location, and Surgical Service were included as categorical values in the regression model. All other variables were included in the final model if the p-value was < 0.05.

SUPPLEMENTAL TABLE 2A. Final factors included in the binary logistic regression models for Mean Arterial Blood Pressure <55 mmHg for > 1 minute.

|  | | Wald | df | P value | OR | 95% CI for OR | |
| --- | --- | --- | --- | --- | --- | --- | --- |
|  |  |  |  |  |  | Lower | Upper |
|  | Provider |  |  |  |  |  |  |
|  | Resident (reference) | 6.337 | 2 | .042 |  |  |  |
|  | Anesthesiologist | 5.706 | 1 | .017 | 1.242 | 1.040 | 1.483 |
|  | CRNA/CAA | .329 | 1 | .566 | 1.029 | .933 | 1.134 |
|  | Location |  |  |  |  |  |  |
|  | Community Hospital (reference) | 41.637 | 2 | <.001 |  |  |  |
|  | Ambulatory Center | 10.405 | 1 | .001 | .768 | .654 | .902 |
|  | Medical Center | 14.261 | 1 | <.001 | 1.201 | 1.092 | 1.320 |
|  | Surgical Service |  |  |  |  |  |  |
|  | Gynecology (reference) | 73.675 | 6 | <.001 |  |  |  |
|  | Neurosurgery | 31.045 | 1 | <.001 | 1.678 | 1.398 | 2.013 |
|  | General/Plastics | 8.065 | 1 | .005 | 1.194 | 1.056 | 1.349 |
|  | Head and Neck | 17.347 | 1 | <.001 | 1.336 | 1.166 | 1.532 |
|  | Orthopedics | 19.296 | 1 | <.001 | 1.298 | 1.156 | 1.459 |
|  | Urology | 5.093 | 1 | .024 | .817 | .686 | .974 |
|  | ACS, Thoracic, Vascular | 13.997 | 1 | <.001 | 1.387 | 1.169 | 1.646 |
|  | Age | 72.723 | 1 | <.001 | 1.010 | 1.008 | 1.012 |
|  | Anesthesia Duration | 351.489 | 1 | <.001 | 1.004 | 1.003 | 1.004 |
|  | Cardiac Arrythmia | 34.921 | 1 | <.001 | 1.403 | 1.254 | 1.570 |
|  | Chronic Pulmonary Disease | 7.601 | 1 | .006 | 1.171 | 1.047 | 1.310 |
|  | Peripheral Vascular Disease | 16.842 | 1 | <.001 | 1.483 | 1.228 | 1.790 |
|  | Constant | 418.650 | 1 | <.001 | .139 |  |  |

Provider, Location, and Surgical Service were included as categorical values in the regression model. All other variables were included in the final model if the p-value was < 0.05.

SUPPLEMENTAL TABLE 2B. Final factors included in the binary logistic regression models for Mean Arterial Blood Pressure <55 mmHg for > 10 minutes.

|  | | Wald | df | p value | OR | 95% CI for OR | |
| --- | --- | --- | --- | --- | --- | --- | --- |
|  |  |  |  |  |  | Lower | Upper |
|  | Provider |  |  |  |  |  |  |
|  | Resident (reference) | 6.096 | 2 | .047 |  |  |  |
|  | Anesthesiologist | 5.266 | 1 | .022 | 1.734 | 1.084 | 2.774 |
|  | CRNA/CAA | .114 | 1 | .736 | 1.043 | .816 | 1.333 |
|  | Location |  |  |  |  |  |  |
|  | Community Hospital (reference) | 9.880 | 2 | .007 |  |  |  |
|  | Ambulatory Center | .009 | 1 | .925 | .977 | .599 | 1.594 |
|  | Medical Center | 7.839 | 1 | .005 | 1.473 | 1.123 | 1.931 |
|  | Surgical Service |  |  |  |  |  |  |
|  | Gynecology (reference) | 36.386 | 6 | <.001 |  |  |  |
|  | Neurosurgery | 7.993 | 1 | .005 | 1.914 | 1.220 | 3.002 |
|  | General/Plastics | 1.375 | 1 | .241 | 1.250 | .861 | 1.816 |
|  | Head and Neck | 10.235 | 1 | .001 | 1.864 | 1.273 | 2.729 |
|  | Orthopedics | 21.244 | 1 | <.001 | 2.225 | 1.584 | 3.127 |
|  | Urology | .308 | 1 | .579 | 1.166 | .679 | 2.002 |
|  | ACS, Thoracic, Vascular | 10.157 | 1 | .001 | 2.037 | 1.315 | 3.154 |
|  | Anesthesia Duration | 118.787 | 1 | <.001 | 1.004 | 1.003 | 1.005 |
|  | Cardiac Arrythmia | 4.525 | 1 | .033 | 1.314 | 1.022 | 1.690 |
|  | Diabetes Complicated | 13.289 | 1 | <.001 | 2.612 | 1.559 | 4.376 |
|  | Constant | 448.193 | 1 | <.001 | .007 |  |  |

Provider, Location, and Surgical Service were included as categorical values in the regression model. All other variables were included in the final model if the p-value was < 0.05.

SUPPLEMENTAL TABLE 2C. Final factors included in the binary logistic regression models for Mean Arterial Blood Pressure <65 mmHg for > 15 minutes.

|  | | Wald | df | p value | OR | 95% CI for OR | |
| --- | --- | --- | --- | --- | --- | --- | --- |
|  |  |  |  |  |  | Lower | Upper |
|  | Provider |  |  |  |  |  |  |
|  | Resident (reference) | 24.819 | 2 | <.001 |  |  |  |
|  | Anesthesiologist | 5.969 | 1 | .015 | 1.245 | 1.044 | 1.484 |
|  | CRNA/CAA | 5.932 | 1 | .015 | .887 | .806 | .977 |
|  | Location |  |  |  |  |  |  |
|  | Community Hospital (reference) | 34.632 | 2 | <.001 |  |  |  |
|  | Ambulatory Center | .397 | 1 | .529 | .951 | .815 | 1.111 |
|  | Medical Center | 25.108 | 1 | <.001 | 1.274 | 1.159 | 1.401 |
|  | Surgical Service |  |  |  |  |  |  |
|  | Gynecology (reference) | 103.353 | 6 | <.001 |  |  |  |
|  | Neurosurgery | 3.981 | 1 | .046 | 1.204 | 1.003 | 1.444 |
|  | General/Plastics | 6.896 | 1 | .009 | 1.177 | 1.042 | 1.329 |
|  | Head and Neck | 16.885 | 1 | <.001 | 1.330 | 1.161 | 1.523 |
|  | Orthopedics | 78.949 | 1 | <.001 | 1.683 | 1.501 | 1.888 |
|  | Urology | 2.189 | 1 | .139 | 1.137 | .959 | 1.347 |
|  | ACS, Thoracic, Vascular | 7.632 | 1 | .006 | 1.274 | 1.073 | 1.512 |
|  | Anesthesia Duration | 467.909 | 1 | <.001 | 1.004 | 1.004 | 1.005 |
|  | Peripheral Vascular Disease | 15.837 | 1 | <.001 | 1.451 | 1.208 | 1.744 |
|  | Constant | 356.309 | 1 | <.001 | .214 |  |  |

Provider, Location, and Surgical Service were included as categorical values in the regression model. All other variables were included in the final model if the p-value was < 0.05.

SUPPLEMENTAL TABLE 3. Final factors included in the binary logistic regression models for all pulmonary complications.

|  | | Wald | df | p value | OR | 95% CI for OR | |
| --- | --- | --- | --- | --- | --- | --- | --- |
|  |  |  |  |  |  | Lower | Upper |
|  | Provider |  |  |  |  |  |  |
|  | Resident (reference) | .916 | 2 | .633 |  |  |  |
|  | Anesthesiologist | .352 | 1 | .553 | .525 | .063 | 4.400 |
|  | CRNA/CAA | .330 | 1 | .566 | 1.179 | .671 | 2.071 |
|  | Location |  |  |  |  |  |  |
|  | Community Hospital (reference) | 1.736 | 2 | .420 |  |  |  |
|  | Ambulatory Center | .125 | 1 | .724 | .687 | .086 | 5.493 |
|  | Medical Center | 1.296 | 1 | .255 | 1.471 | .757 | 2.856 |
|  | Surgical Service |  |  |  |  |  |  |
|  | Gynecology (reference) | 18.494 | 6 | .005 |  |  |  |
|  | Neurosurgery | 5.343 | 1 | .021 | 11.457 | 1.449 | 90.592 |
|  | General/Plastics | 6.068 | 1 | .014 | 12.456 | 1.674 | 92.669 |
|  | Head and Neck | 3.509 | 1 | .061 | 7.261 | .912 | 57.780 |
|  | Orthopedics | 1.951 | 1 | .162 | 4.335 | .554 | 33.935 |
|  | Urology | 1.214 | 1 | .270 | 3.910 | .346 | 44.209 |
|  | ACS, Thoracic, Vascular | 6.209 | 1 | .013 | 13.279 | 1.737 | 101.539 |
|  | Anesthesia Duration | 30.968 | 1 | <.001 | 1.004 | 1.003 | 1.005 |
|  | ASA Score | 24.815 | 1 | <.001 | 2.526 | 1.754 | 3.637 |
|  | Cardiac Arrythmia | 68.156 | 1 | <.001 | 7.656 | 4.722 | 12.413 |
|  | Chronic Pulmonary Disease | 6.531 | 1 | .011 | 1.842 | 1.153 | 2.942 |
|  | Congestive Heart Failure | 4.743 | 1 | .029 | 1.783 | 1.060 | 2.999 |
|  | Constant | 96.054 | 1 | <.001 | .000 |  |  |

Provider, Location, and Surgical Service were included as categorical values in the regression model. All other variables were included in the final model if the p-value was < 0.05.

SUPPLEMENTAL TABLE 4. Final factors included in the binary logistic regression models for all cardiac complications.

|  | | Wald | df | p value | OR | 95% CI for OR | |
| --- | --- | --- | --- | --- | --- | --- | --- |
|  |  |  |  |  |  | Lower | Upper |
|  | Provider |  |  |  |  |  |  |
|  | Resident (reference) | 1.131 | 2 | .568 |  |  |  |
|  | Anesthesiologist | .000 | 1 | .990 | .000 | .000 |  |
|  | CRNA/CAA | 1.131 | 1 | .287 | 1.704 | .638 | 4.549 |
|  | Location |  |  |  |  |  |  |
|  | Community Hospital (reference) | 3.311 | 2 | .191 |  |  |  |
|  | Ambulatory Center | .000 | 1 | .989 | .000 | .000 |  |
|  | Medical Center | 3.311 | 1 | .069 | 2.787 | .924 | 8.405 |
|  | Surgical Service |  |  |  |  |  |  |
|  | Gynecology (reference) | 3.478 | 6 | .747 |  |  |  |
|  | Neurosurgery | .000 | 1 | .985 | .000 | .000 |  |
|  | General/Plastics | .000 | 1 | .985 | .000 | .000 |  |
|  | Head and Neck | .000 | 1 | .987 | .000 | .000 |  |
|  | Orthopedics | .000 | 1 | .985 | .000 | .000 |  |
|  | Urology | .000 | 1 | .985 | .000 | .000 |  |
|  | ACS, Thoracic, Vascular | .000 | 1 | .985 | .000 | .000 | . |
|  | ASA Score | 11.036 | 1 | <.001 | 2.853 | 1.537 | 5.297 |
|  | Cardiac Arrythmia | 8.946 | 1 | .003 | 2.974 | 1.456 | 6.075 |
|  | Diabetes Uncomplicated | 16.990 | 1 | <.001 | 4.291 | 2.147 | 16.990 |
|  | Coronary Artery Disease | 25.847 | 1 | <.001 | 6.887 | 3.273 | 25.847 |
|  | Constant | .001 | 1 | .973 | .000 |  |  |

Provider, Location, and Surgical Service were included as categorical values in the regression model. All other variables were included in the final model if the p-value was < 0.05.

SUPPLEMENTAL TABLE 5. Final factors included in the binary logistic regression models for myocardial infarction.

|  | | Wald | df | p value | OR | 95% CI for OR | |
| --- | --- | --- | --- | --- | --- | --- | --- |
|  |  |  |  |  |  | Lower | Upper |
|  | Provider |  |  |  |  |  |  |
|  | Resident (reference) | 1.565 | 2 | .457 |  |  |  |
|  | Anesthesiologist | .743 | 1 | .389 | 2.134 | .381 | 11.967 |
|  | CRNA/CAA | 1.371 | 1 | .242 | 1.520 | .754 | 3.061 |
|  | Location |  |  |  |  |  |  |
|  | Community Hospital (reference) | 2.962 | 2 | .227 |  |  |  |
|  | Ambulatory Center | .000 | 1 | .990 | .000 | .000 | . |
|  | Medical Center | 2.962 | 1 | .085 | 2.239 | .894 | 5.606 |
|  | Surgical Service |  |  |  |  |  |  |
|  | Gynecology (reference) | 18.309 | 6 | .006 |  |  |  |
|  | Neurosurgery | 2.469 | 1 | .116 | .281 | .058 | 1.368 |
|  | General/Plastics | .548 | 1 | .459 | .710 | .287 | 1.756 |
|  | Head and Neck | 4.489 | 1 | .034 | .229 | .058 | .895 |
|  | Orthopedics | .329 | 1 | .566 | .774 | .323 | 1.857 |
|  | Urology | .083 | 1 | .773 | .820 | .212 | 3.171 |
|  | ACS, Thoracic, Vascular | 2.847 | 1 | .092 | 2.164 | .883 | 5.307 |
|  | Anesthesia Duration | 30.155 | 1 | <.001 | 1.005 | 1.003 | 1.007 |
|  | Cardiac Arrythmia | 30.445 | 1 | <.001 | 4.498 | 2.637 | 7.673 |
|  | Coronary Artery Disease | 7.317 | 1 | .007 | 2.275 | 1.254 | 4.128 |
|  | Constant | 127.843 | 1 | <.001 | .000 |  |  |

Provider, Location, and Surgical Service were included as categorical values in the regression model. All other variables were included in the final model if the p-value was < 0.05.

SUPPLEMENTAL TABLE 6. Final factors included in the binary logistic regression models for mortality.

|  | | Wald | df | p value | OR | 95% CI for OR | |
| --- | --- | --- | --- | --- | --- | --- | --- |
|  |  |  |  |  |  | Lower | Upper |
|  | Provider |  |  |  |  |  |  |
|  | Resident (reference) | 1.484 | 2 | .476 |  |  |  |
|  | Anesthesiologist | .000 | 1 | .995 | .000 | .000 | . |
|  | CRNA/CAA | 1.484 | 1 | .223 | 1.756 | .710 | 4.345 |
|  | Location |  |  |  |  |  |  |
|  | Community Hospital (reference) | .049 | 2 | .976 |  |  |  |
|  | Ambulatory Center | .000 | 1 | .998 | .000 | .000 | . |
|  | Medical Center | .049 | 1 | .825 | 1.099 | .475 | 2.541 |
|  | Surgical Service |  |  |  |  |  |  |
|  | Gynecology (reference) | 11.189 | 6 | .083 |  |  |  |
|  | Neurosurgery | 2.790 | 1 | .095 | 6.242 | .728 | 53.521 |
|  | General/Plastics | .614 | 1 | .433 | 2.325 | .282 | 19.192 |
|  | Head and Neck | .489 | 1 | .484 | 2.264 | .229 | 22.379 |
|  | Orthopedics | .071 | 1 | .790 | 1.338 | .157 | 11.402 |
|  | Urology | 1.687 | 1 | .194 | 4.371 | .472 | 40.477 |
|  | ACS, Thoracic, Vascular | 1.973 | 1 | .160 | 4.417 | .556 | 35.098 |
|  | Age | 16.408 | 1 | <.001 | 1.054 | 1.027 | 1.081 |
|  | ASA Score | 8.280 | 1 | .004 | 2.315 | 1.307 | 4.101 |
|  | Hypertension Complicated | 11.706 | 1 | <.001 | 3.065 | 1.614 | 5.822 |
|  | Pulmonary Complications All | 21.057 | 1 | <.001 | 8.317 | 3.365 | 20.555 |
|  | AKI (any grade) | 12.892 | 1 | <.001 | 3.421 | 1.748 | 6.696 |
|  | Constant | 62.051 | 1 | <.001 | .000 |  |  |

Provider, Location, and Surgical Service were included as categorical values in the regression model. All other variables were included in the final model if the p-value was < 0.05.

SUPPLEMENTAL TABLE 7. Final factors included in the binary logistic regression models for AKI.

|  | | Wald | df | p value | OR | 95% CI for OR | |
| --- | --- | --- | --- | --- | --- | --- | --- |
|  |  |  |  |  |  | Lower | Upper |
|  | Provider |  |  |  |  |  |  |
|  | Resident (reference) | 1.893 | 2 | .388 |  |  |  |
|  | Anesthesiologist | .239 | 1 | .625 | 1.169 | .625 | 2.187 |
|  | CRNA/CAA | 1.876 | 1 | .171 | 1.224 | .917 | 1.634 |
|  | Location |  |  |  |  |  |  |
|  | Community Hospital (reference) | 6.014 | 2 | .049 |  |  |  |
|  | Ambulatory Center | 4.467 | 1 | .035 | .725 | .538 | .977 |
|  | Medical Center | 2.084 | 1 | .149 | .227 | .030 | 1.700 |
|  | Surgical Service |  |  |  |  |  |  |
|  | Gynecology (reference) | 42.901 | 6 | <.001 |  |  |  |
|  | Neurosurgery | 19.100 | 1 | <.001 | .261 | .143 | .477 |
|  | General/Plastics | 2.036 | 1 | .154 | .745 | .496 | 1.117 |
|  | Head and Neck | 2.712 | 1 | .100 | .659 | .402 | 1.082 |
|  | Orthopedics | 1.241 | 1 | .265 | .800 | .540 | 1.185 |
|  | Urology | 3.433 | 1 | .064 | 1.594 | .973 | 2.610 |
|  | ACS, Thoracic, Vascular | .482 | 1 | .488 | 1.162 | .760 | 1.778 |
|  | Age | 12.455 | 1 | <.001 | 1.013 | 1.006 | 1.020 |
|  | Anesthesia Duration | 11.893 | 1 | <.001 | 1.001 | 1.001 | 1.002 |
|  | ASA Score | 15.679 | 1 | <.001 | 1.440 | 1.202 | 1.725 |
|  | Cardiac Arrhythmias | 12.186 | 1 | <.001 | 1.503 | 1.196 | 1.889 |
|  | Congestive Heart Failure | 7.030 | 1 | .008 | 1.519 | 1.115 | 2.070 |
|  | Renal Insufficiency | 34.095 | 1 | <.001 | 2.175 | 1.676 | 2.824 |
|  | Constant | 146.787 | 1 | <.001 | .011 |  |  |

Provider, Location, and Surgical Service were included as categorical values in the regression model. All other variables were included in the final model if the p-value was < 0.05.

SUPPLEMENTAL TABLE 8A. General Linear Regression Model for the Estimated Marginal Mean for Length of Stay for Providers

| Provider | Estimated Mean | Std. Error | 95% Confidence Interval | |
| --- | --- | --- | --- | --- |
|  |  |  | Lower Bound | Upper Bound |
| Anesthesiologist | 2.005 | .753 | .530 | 3.481 |
| CRNA/CAA | 2.024 | .078 | 1.870 | 2.177 |
| Resident | 1.968 | .328 | 1.326 | 2.610 |

General Linear Model in SPSS for the categorical variables of provider group and location with inclusion of the following significant covariates from TABLE 1 – Age, Total OR time, Body Mass Index, arterial line placement, surgical service, ASA status, Race, Cardiac Arrhythmia, Chronic Pulmonary Disease, Hypertension Complicated, Hypertension Uncomplicated, Peripheral Vascular Disorders, and Renal Insufficiency

SUPPLEMENTAL TABLE 8B. Pairwise Comparisons for the Estimated Marginal Mean for Length of Stay for Providers

| (I) Provider | (J) Provider | Mean Difference (I-J) | Std. Error | P value | 95% Confidence Interval for Difference | |
| --- | --- | --- | --- | --- | --- | --- |
|  |  |  |  |  | Lower Bound | Upper Bound |
| Anesthesiologist | CRNA/CAA | -.018 | .756 | 1.000 | -1.829 | 1.793 |
|  | Resident | .038 | .821 | 1.000 | -1.927 | 2.002 |
| CRNA/CAA | Anesthesiologist | .018 | .756 | 1.000 | -1.793 | 1.829 |
|  | Resident | .056 | .336 | 1.000 | -.749 | .861 |
| Resident | Anesthesiologist | -.038 | .821 | 1.000 | -2.002 | 1.927 |
|  | CRNA/CAA | -.056 | .336 | 1.000 | -.861 | .749 |

Based on estimated marginal means

P value adjustment for multiple comparisons: Bonferroni.

SUPPLEMENTAL TABLE 8C. Estimated Marginal Mean for Length of Stay Stratified by Location and Provider

| Location | Provider | Estimated Mean | Std. Error | 95% Confidence Interval | |
| --- | --- | --- | --- | --- | --- |
|  |  |  |  | Lower Bound | Upper Bound |
| Community Hospital | Anesthesiologist | 1.520 | .207 | 1.113 | 1.926 |
|  | CRNA/CAA | 1.769 | .117 | 1.539 | 2.000 |
|  | Resident | 1.971 | .599 | .796 | 3.145 |
| Ambulatory Center | Anesthesiologist | 1.901 | 2.026 | -2.071 | 5.873 |
|  | CRNA/CAA | 1.367 | .189 | .997 | 1.738 |
|  | Resident | 1.124 | .767 | -.379 | 2.628 |
| Medical Center | Anesthesiologist | 2.596 | .974 | .688 | 4.505 |
|  | CRNA/CAA | 2.934 | .080 | 2.777 | 3.091 |
|  | Resident | 2.808 | .138 | 2.538 | 3.078 |
|  | | | | | |

General Linear Model in SPSS for the categorical variables of provider group and location with inclusion of the following significant covariates from TABLE 1 – Age, Total OR time, Body Mass Index, arterial line placement, surgical service, ASA status, Race, Cardiac Arrhythmia, Chronic Pulmonary Disease, Hypertension Complicated
